# Enterotoxigenic *Escherichia coli* and *Vibrio cholerae* Diarrhea, Bangladesh, 2004

**DOI:** 10.3201/eid1107.041266

**Published:** 2005-07

**Authors:** Firdausi Qadri, Ashraful I. Khan, Abu Syed G. Faruque, Yasmin Ara Begum, Fahima Chowdhury, Gopinath B. Nair, Mohammed A. Salam, David A. Sack, Ann-Mari Svennerholm

**Affiliations:** * International Centre for Diarrhoeal Disease Research, Bangladesh, Dhaka, Bangladesh;; † Göteborg University, Göteborg, Sweden

**Keywords:** Diarheal epidemic, ETEC, V. Cholera

## Abstract

Flooding in Dhaka in July 2004 caused epidemics of diarrhea. Enterotoxigenic *Escherichia coli* (ETEC) was almost as prevalent as *Vibrio cholerae* O1 in diarrheal stools. ETEC that produced heat-stable enterotoxin alone was most prevalent, and 78% of strains had colonization factors. Like *V. cholerae* O1, ETEC can cause epidemic diarrhea.

In July 2004, Bangladesh experienced devastating floods, which also affected the capital, Dhaka, and outbreaks of diarrheal diseases occurred throughout the city. As a result, a steep increase was seen in patient admissions, which reached epidemic numbers around July 20, when >350 patients were admitted every day to the hospital of the International Centre for Diarrhoeal Disease Research, Bangladesh (ICDDR,B). During the peak period, >700 patients were seen per day, and the total number seen during the epidemic was >17,000.

Diarrhea caused by enterotoxigenic *Escherichia coli* (ETEC) is highly prevalent in young children in developing countries as well as in travelers to these areas (1). In Bangladesh, *Vibrio cholerae* is the bacterial pathogen that most frequently necessitates hospitalization (2). ETEC is also commonly isolated from patients seeking treatment in hospitals (3–5), but it is not actively screened for during natural disasters. However, reports have suggested that ETEC, in addition to cholera, is a predominant cause of diarrhea in Bangladesh (6,7). Since ETEC spreads though contaminated water and food (8,9), we analyzed diarrheal stools for this pathogen to assess the prevalence of ETEC during the epidemic.

ETEC causes diarrhea by producing different combinations of the heat labile (LT) or heat stable (ST) enterotoxins and 1 or more of at least 22 different colonization factors, which contribute to the virulence of the pathogen (10). Since genes for these factors are predominantly present on plasmids, which may be lost on storage, we tested for phenotypic expression of these factors by using freshly cultured isolates. For this purpose, diarrheal stools were collected from patients in a 2% systematic routine surveillance system; every 50th patient attending the hospital is routinely screened for *V. cholerae*, *Shigella* spp., and *Salmonella* spp (4). at the Clinical Research and Service Centre of the ICDDR, B. The study was approved by the institutional review board of ICDDR,B.

## The Study

Only samples negative for *V. cholerae* were tested for ETEC, starting from July 20, 2004, when the patient numbers increased at the ICDDR,B hospital for ≈6 weeks, until the patient numbers decreased and the floods had receded. Information, including age, sex, fever, vomiting, dehydration status, and related clinical features, was also collected from patients. For ETEC surveillance, we used lactose-fermenting *E. coli* colonies cultured on MacConkey agar plates that had been cultured from fresh stool specimens (4). Six lactose-fermenting individual colonies of *E. coli* were tested for the presence of LT, ST, and colonization factors. Detection of LT and ST was carried out with ganglioside GM1 enzyme-linked immunosorbent assays (4). The colonies that tested positive for the toxins were also plated onto colonization factor antigen (CFA) agar plates with and without bile salts for testing colonization factors (4). Trypticase soy agar containing 5% sheep blood (TSA) was used to test for the colonization factor CS21 (5).

The strains were cultured at 37°C overnight; those grown on CFA agar without bile were tested for colonization factors CFA/1, CSI, CS2, CS3, CS4, and CS6, and those on CFA agar plus bile were tested for CS5, CS7, CS17, CS8, CS12, and CS14 (4). Those strains grown on TSA were tested for CS21 only (5). Of the patients included in this study, 67% had severe-to-moderate dehydration; of these, 51% were children <5 years of age, while 39% were >15 years of age. They were treated for diarrhea with oral (61%) or intravenous (39%) rehydration therapy and other medications as needed.

Of 350 stool specimens tested during the epidemic, 78 (22.2%) were positive for *V. cholerae* O1 (22 Ogawa and 56 Inaba serotype), and 63 (18.0%) were positive for ETEC. *Shigella* spp. (3.4%, n = 11) and *Salmonella* spp. (1.7%, n = 5) were seen at lower rates. Children with ETEC diarrhea were negative for *V. cholerae* O1 as well as *Shigella* spp. and *Salmonella* spp. We did not test *V. cholerae*–positive samples for ETEC and therefore cannot rule out possible concomitant infection with ETEC in these 78 cholera patients (4).

Isolation of ETEC and *V. cholerae* O1 remained high throughout the epidemic (Figure), and during 1 week, comparable numbers of ETEC and *V. cholerae* were isolated from stools of patients. We compared demographic and clinical features of patients with ETEC and *V. cholerae* infections (Table 1). Most patients with ETEC diarrhea were <2 years of age (56%) or >15 years of age (36%) (median 1.5 years), whereas those with *V. cholerae* O1 infection were mostly >5 years of age (median age 15.5 years). Although more cholera patients had severe dehydration (60%), 22% of the patients with ETEC diarrhea also had severe dehydration (p<0.001). Intravenous rehydration was needed for both ETEC- and *V. cholerae*–infected patients, but it was more frequently used in the latter.

**Figure:**
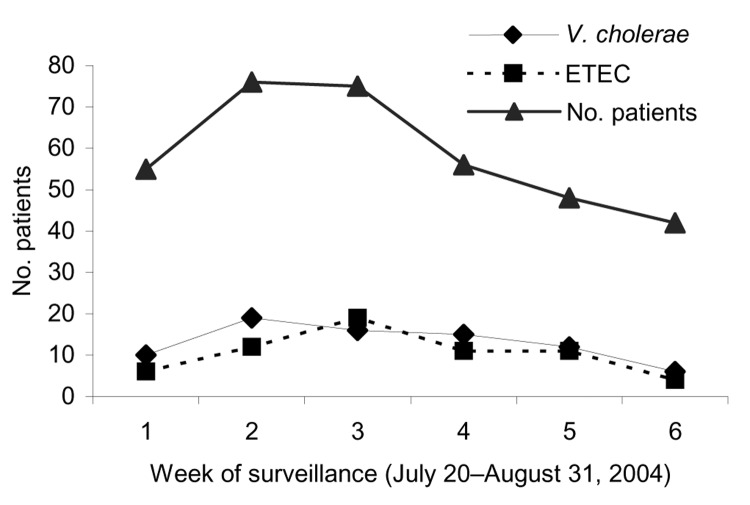
Weekly distribution of patients with *Vibrio cholerae* O1 or enterotoxigenic *Escherichia coli* (ETEC) infections during the study period from July 20 to August 31, 2004. The total number of patients who underwent stool analyses at the treatment center each week during the diarrheal epidemic is also shown.

**Table 1:** Characteristics of the patients with enterotoxigenic *Escherichia coli* (ETEC) and *Vibrio cholerae* O1 infection during the diarrheal epidemic, July–August 2004, Bangladesh

Parameter	No. ETEC* (n = 63) (%)	No. V. cholerae* (n = 78) (%)	All patients† (N = 350) (%)
Age			
≤2 y	35 (56)	9 (12)	159 (45)
3–4 y	3 (5)	9 (12)	22 (6)
5–15 y	2 (3)	21 (27)	33 (9)
>15 y	23 (36)	39 (50)	136 (39)
Median (mo)	18	186	48
Range (mo)	1.9–600.0	4.9–780.0	0.67–960
Sex			
Male	35 (56)	41 (53)	198 (57)
Female	28 (44)	37 (47)	152 (43)
Dehydration status			
No sign	29 (46)	4 (5)	115 (33)
Some	20 (32)	27 (35)	133 (38)
Severe	14 (22)	47 (60)	102 (29)
Intravenous rehydration needed	18 (29)	56 (72)	137 (39)

*No. patients infected with respective bacterial pathogens seen at the International Centre for Diarrhoeal Disease Research, Bangladesh treatment center during the epidemic. †Total patients with specimens tested during the study period.

With regard to toxin profile, ETEC expressing ST alone was the most common (67%), followed by strains producing both ST and LT (19%) and LT alone (14%). Dominance of the ST-expressing ETEC has been documented earlier during seasonal outbreaks and epidemics in Bangladesh (4) and in Egypt and the Middle East (11,12). Patients infected with the different toxin phenotypes of ETEC had dehydration status ranging from severe to none, although no significant association was seen between toxin phenotype and degree of dehydration.

A high proportion of the ETEC strains (78%) expressed 1 or more colonization factors (Table 2), a much higher frequency than that seen in other hospital or community-based studies (10,12). In earlier studies in Bangladesh, we found 56% of strains positive for these colonization factors (4). In the present study, ≈92% of ST/LT-, 79% of ST-, and 56% of LT-expressing ETEC expressed 1 or more colonization factors. CFA/I was the most common phenotype, followed by the strains expressing CS4 + CS6 or CS5 + CS6, followed by others. Thus, most of the colonization factor types were those known to be present in clinical strains and those that have previously been isolated from hospitalized patients (4,5). These antigens have been given priority for designing vaccines to protect against a wide range of colonization factors (10). In addition, 3 strains co-expressed CS21, a type IV pilus antigen (4). Of these, 2 strains expressed CFA/I and CS21, and 1 was positive for CS1, CS3, and CS21.

**Table 2:** Colonization factor (CF) types of enterotoxigenic *Escherichia coli* (ETEC) isolated from patients during diarrhea epidemic, Bangladesh*

Toxin produced	CF type(s) produced	No. isolates (%)
ST (n = 42)	CFA/I	9 (21.4)
	CFA/I, CS21	1 (2.38)
	CS1 + CS3, CS21	1 (2.38)
	CS4 + CS6	7 (16.67)
	CS5 + CS6	7 (16.67)
	CS6	6 (14.29)
	CS14	1 (2.38)
	CS17	1 (2.38)
LT/ST (n = 12)	CFA/	1 (8.33)
	CFA/I, CS21	1 (8.33)
	CS1 + CS3	2 (16.67)
	CS2 + CS3	1 (8.33)
	CS4 + CS6	2 (16.67)
	CS5 + CS6	3 (25.00)
	CS14	1 (8.33)
LT (n = 9)	CS7	3 (33.3)
	CS6 + CS8	1 (11.1)
	CS17	1 (11.1)

*Of the 63 ETEC strains isolated, 78% were positive for 1 or more of the 12 CFs tested. Those that were positive are shown above. ST, heat stable; LT, heat labile

We used 13 colonization factor–specific monoclonal antibodies in testing; however, >22 colonization factors have been described, not all of which could be tested in this study. In addition, although precautions were taken to rule out the loss of phenotypic properties of colonization factors, some may have been lost on culture. By using polymerase chain reaction or DNA hybridization procedures, more colonization factor–specific genes and those that have undergone phenotypic changes could have been detected (13).

## Conclusions

We hypothesize that contaminated water during floods can be a cause of ETEC diarrhea. Flood waters may be contaminated by sewage, increasing transmission by the fecal-oral route. Our recent studies have also shown that ETEC can be isolated relatively frequently from surface water samples in Bangladesh (14).

Although diarrhea can be prevented by improving water quality, sanitation, and overall hygiene, these improvements will not be possible in the near future in densely populated areas with limited resources. Thus, developing vaccines that can prevent such epidemics is a goal. Such vaccines should include at least the most prevalent colonization factors, such as those found on the ETEC strains we isolated, to provide protection against the virulent, colonization factor–expressing, ST-positive ETEC strains.

This article emphasizes that ETEC can be a major source of acute watery diarrhea in epidemics caused by floods. This report is the first to show that during waterborne natural disasters, ETEC can also cause dehydrating diarrhea severe enough to require clinical care and, in many instances, intravenous rehydration. During epidemics, focus on ETEC should be on pediatric patients <2 years of age, since ETEC was the most prevalent bacterial enteropathogen identified in this age group. The treatment strategy should be designed accordingly, since ETEC strains are becoming increasingly resistant to erythromycin (15), the drug usually used for young children with acute watery diarrhea, irrespective of diagnosis.
